# Genomic and transcriptomic analyses provide insights into valuable fatty acid biosynthesis and environmental adaptation of yellowhorn

**DOI:** 10.3389/fpls.2022.991197

**Published:** 2022-09-06

**Authors:** Qiang Liang, Jian Ning Liu, Hongcheng Fang, Yuhui Dong, Changxi Wang, Yan Bao, Wenrui Hou, Rui Zhou, Xinmei Ma, Shasha Gai, Lichang Wang, Shouke Li, Ke Qiang Yang, Ya Lin Sang

**Affiliations:** ^1^College of Forestry, Shandong Agricultural University, Tai’an, Shandong, China; ^2^Worth Agricultural Development Co. Ltd., Weifang, China; ^3^State Forestry and Grassland Administration Key Laboratory of Silviculture in Downstream Areas of the Yellow River, Shandong Agricultural University, Tai’an, Shandong, China; ^4^Shandong Taishan Forest Ecosystem Research Station, Shandong Agricultural University, Tai’an, Shandong, China

**Keywords:** yellowhorn (*Xanthoceras sorbifolium*), haplotype-resolved, genome assembly, transcriptome, fatty acids biosynthesis, adaptation

## Abstract

Yellowhorn (*Xanthoceras sorbifolium*) is an oil-bearing tree species growing naturally in poor soil. The kernel of yellowhorn contains valuable fatty acids like nervonic acid. However, the genetic basis underlying the biosynthesis of valued fatty acids and adaptation to harsh environments is mainly unexplored in yellowhorn. Here, we presented a haplotype-resolved chromosome-scale genome assembly of yellowhorn with the size of 490.44 Mb containing scaffold N50 of 34.27 Mb. Comparative genomics, in combination with transcriptome profiling analyses, showed that expansion of gene families like long-chain acyl-CoA synthetase and ankyrins contribute to yellowhorn fatty acid biosynthesis and defense against abiotic stresses, respectively. By integrating genomic and transcriptomic data of yellowhorn, we found that the transcription of *3-ketoacyl-CoA synthase* gene *XS04G00959* was consistent with the accumulation of nervonic and erucic acid biosynthesis, suggesting its critical regulatory roles in their biosynthesis. Collectively, these results enhance our understanding of the genetic basis underlying the biosynthesis of valuable fatty acids and adaptation to harsh environments in yellowhorn and provide foundations for its genetic improvement.

## Introduction

Plant oil is the primary source of edible fatty acid in the human diet and provides a natural and renewable feedstock for diverse industrial applications (Sharma et al., 2012; Abiodun, 2017). Global plant oil production increased from approximately 161.6 million metric tons in 2012 to 213.2 million in 2022 (Statista, 2022). However, due to the decreases in agricultural land and the rapid climate changes, it has become challenging to meet the growing demands for edible plant oil (Huang et al., 2016; Balbino, 2017; Yang et al., 2018; Chen et al., 2020). To address this need, it is essential to utilize plant species with high oil production and solid environmental adaptation (Bailey-Serres et al., 2019; Ortiz et al., 2020). Among many plant species, yellowhorn (*Xanthoceras sorbifolium*), a solid woody oleiferous plant, is an ideal material for meeting the demand (Ruan et al., 2017).

Yellowhorn, the sole member of the *Xanthoceras* (Sapindaceae), is distributed widely in northern China and adapted to harsh environmental conditions in the warm temperate zone (Group, 2016; Zhao et al., 2021). This species exhibits strong resistance to drought, salt, and cold stress and has been considered a promising ecological restoration species (Ruan et al., 2017). Yellowhorn produces capsular fruits from hermaphrodite. The seed of yellowhorn is rich in oil, which has been classified as edible oil by the National Health Commission of the People’s Republic of China.^1^ Yellowhorn seed oil is considered one of the better among 38 plant species producing nervonic acid (Yu et al., 2017; Liu F. et al., 2021). Compared with other oleaginous woody plants producing nervonic acid, such as *Malania oleifera*, *Ximenia caffra*, *Acer truncatum*, yellowhorn has higher seed oil content (52.7–58.0%), lower nervonic acid (3.46–4.37%), and moderate erucic acid (8.99–9.83%) content of total fatty acid (Liang et al., 2021; Liu F. et al., 2021). Nervonic acid is crucial for nerve myelin synthesis, which is one of the fundamental components of the nerve and brain (Poulos, 1995; Merrill et al., 1997; Martínez and Mougan, 1998). It has been found that nervonic acid is essential for developing the human nervous system and can be used to prevent and treat various neurodegenerative diseases (Kageyama et al., 2021; Song et al., 2022; Terluk et al., 2022). However, long-term exposure to excessive levels of erucic acid can reduce the β-oxidation of erucic acid in animal myocardia, leading to lipid deposition and tissue damage (Bremer and Norum, 1982). According to the European Food Safety Authority, tolerable levels of daily erucic acid intake are 7 mg/kg body weight (EFSA Panel on Contaminants in the Food Chain [CONTAM], 2016). Then the European Union Commission Regulation 2019/1870 sets the maximum level for erucic acid in vegetable oils as 20.0 g/kg, except for camelina oil, mustard oil, and borage oil, in which the maximum level of erucic acid is 50.0 g/kg. Thus, it is desirable to domesticate and breed yellowhorn varieties that produce increased levels of nervonic acid and decreased levels of erucic acid.

The biosynthesis of nervonic and erucic acid consists of two spatially separated steps. The *de novo* fatty acid synthesis occurs in plastids and gives rise to C18:1-CoA, while very long-chain unsaturated fatty acid (VLCFA) elongation at the endoplasmic reticulum (ER) produces nervonic and erucic acid (Wang P. et al., 2022). The latter was catalyzed by 3-ketoacyl-CoA synthase (KCS), 3-ketoacyl-CoA reductase (KCR), 3-hydroxacyl-CoA dehydratase (HCD), and *trans*-2,3-enoyl-CoA reductase (ECR). Of the four enzymes, KCS is the rate-limiting regulator (Millar and Kunst, 1997; Joubès et al., 2008; Haslam and Kunst, 2013; Wang Y. et al., 2022). KCR, HCD, and ECR are expressed and function in all tissues with fatty acid biosynthesis (Zheng et al., 2005; Paul et al., 2006; Bach et al., 2008; Beaudoin et al., 2009; Haslam and Kunst, 2013). KCS thus determines the tissue specificity of VLCFA elongation. However, the molecular mechanism underlying nervonic acid and erucic acid biosynthesis of yellowhorn is largely unelucidated.

High-level genome assembly is essential for studying the mechanisms of economically important traits and subsequent genetic modifications. Previously, other teams and we reported the yellowhorn genome assembly (Bi et al., 2019; Liang et al., 2019; Liu H. et al., 2021). In this study, we re-assembled the yellowhorn genome at the haplotype-resolved level. Based on the new assembly, genomic and transcriptomic analyses were performed on gene families involved in fatty acid biosynthesis and stress response. The results provided new clues for understanding the molecular mechanisms of environmental adaptation and valuable fatty acid biosynthesis of yellowhorn.

## Materials and methods

### Plant materials

The yellowhorn cultivar ‘Shanyou 1’ (released No.: Lu S-SV-XS-021-2020, designated as ‘WF18’ previously) was employed as plant material and planted in loam soil in 2012 at the yellowhorn orchard of the Forestry Experimental Station of Shandong Agricultural University (117°8′58′′ E, 36°10′16′′N) with orchard management as previously described (Liang et al., 2019, 2021). The seed sac filled with the embryo on June 5, 2017 (50 days after flowering, DAF), and the capsules matured on July 11, 2017 (86 DAF). Therefore, we measured oil accumulation and fatty acid content every 6 days from 50 to 86 DAF. Three capsules were picked from the randomly selected infructescence at 50, 56, 62, 68, 74, 80, and 86 DAF, respectively. The kernel was stripped from each seed immediately, flash-frozen in liquid nitrogen, and then stored at −80°C. Fresh, healthy leaves were collected from each accession for DNA extraction. The high-quality genomic DNA was extracted using the NucleoSpin Plant II (MachereyeNagel, Düren, Germany) according to the recommended procedure by the Kit.

### Genome assembly

The genomic DNA sequencing datasets of PacBio Sequel long reads, Illumina short reads, and Hi-C reads were used for the haplotype-resolved assembly of yellowhorn cultivar ‘Shanyou 1.’ The haplotype-resolved genome assembly was constructed with FALCON v 1.8.1 (Chin et al., 2016) and FALCON-Unzip^2^ using our previous datasets, which included PacBio Sequel long reads and Illumina short reads (Liang et al., 2019). Briefly, pre-assembly (error correction) was performed based on all-by-all alignments of the subreads (length > 5 kb) using the FALCON HPCdaligner module. Preassembled, error-corrected reads longer than eight kbp were selected for final assembly with the Overlap-Layout-Consensus algorithm. Next, based on the draft FALCON assembly, the FALCON-Unzip assembler was applied to separate haplotype-specific contigs, generating primary contigs (phased genome assembly) and fragmented haplotigs (alternate haplotypes). The genome assembly was then polished, firstly with the haplotype-phased reads using the FALCON-Unzip pipeline, secondly with the PacBio long reads using the Arrow algorithm in the GenomicConsensus package v2.3.3,^3^ and finally with the Illumina short reads using Pilon v1.23 (Walker et al., 2014). The polished primary contigs and the Hi-C reads were used to construct chromosome-length scaffolds by applying the 3D-DNA pipeline v180922 (Dudchenko et al., 2017). For this purpose, duplication-free Hi-C contact maps were generated by analyzing kilobase resolution Hi-C data with the Juicer pipeline v1.5 (Durand et al., 2016). The Hi-C contacts were put into 3D-DNA software; then the chromosome-length scaffolds were constructed using default parameters. The genome scaffolds were then manually refined using Juicebox Assembly Tools v1.8.8. The refined Hi-C contacts were selected and clustered into a chromosome-scale assembly using the 3D-DNA pipeline. Finally, the refined chromosome-length scaffolds of the assembly were elaborately optimized with gap filling using LR_Gapcloser v1.1 (Xu et al., 2019) and subsequent polished three rounds using Pilon software. The accuracy of the assembly was evaluated by identifying and calculating the percentages of homogeneous variations using GATK v3.8 (McKenna et al., 2010) based on Illumina short reads. The completeness of the assembly was assessed against the embryophyta_odb10 lineage dataset (1,614 BUSCO groups) using BUSCO v5.0.0 (Seppey et al., 2019), with the parameters “-m genome -c 20-sp arabidopsis.”

### Genome annotation

An integrated *de novo* and sequence homology-based modeling pipeline was used to identify repetitive sequences. First, RepeatModeler v2.0.1,^4^ RepeatScout v1.0.5 (Price et al., 2005), LTR_Finder v1.07 (Xu and Wang, 2007), MITE-Hunter (Han and Wessler, 2010), and PILER (Edgar and Myers, 2005) were used to predict repetitive sequences. The predicted repetitive elements were classified using PASTEClassifier v2.0 (Hoede et al., 2014) and then combined with the repetitive DNA elements in Repbase v25.01 (Bao et al., 2015) to produce a repeat library. The repetitive elements in the assembly were further identified and annotated using RepeatMasker v4.1.0 (Chen, 2004) against this constructed repeat library.

Protein-coding gene identification and annotation were performed as previously described (Liang et al., 2019). The gene prediction programs GeneMark-ES/ET/EP v4.62 (Lomsadze et al., 2005), Augustus v3.0 (Stanke et al., 2008), and SNAP v2006-07-28 (Korf, 2004) were used for *ab initio* gene prediction based on the soft-masked genome assembly. Exonerate v2.2.0 (Slater and Birney, 2005) was used to predict genes based on protein homology by aligning the peptides of several model plant species, including *Arabidopsis thaliana* TAIR10 (Lamesch et al., 2012), *Oryza sativa* IRGSP-1.0 (Kawahara et al., 2013; Sakai et al., 2013), *Populus trichocarpa* v3 (Tuskan et al., 2006), *Solanum lycopersicum* SL3.0 (Tomato Genome Consortium, 2012), *Vitis vinifera* IGGP_12x (Jaillon et al., 2007), and *Glycine max* v2.1 (Schmutz et al., 2010) against the genome assembly. Gene structures were modeled using PASA v2.3.3 (Haas et al., 2003) based on the genome alignments of the *de novo* transcripts assembled by Trinity v2.8.4 (Grabherr et al., 2011). The final gene models in the assembly were generated using MAKER v 2.31.10 (Campbell et al., 2014) by integrating the *ab initio* and homology gene predictions. Genes were functionally annotated using the InterProScan v5.33 pipeline against InterPro database v72.0 (Mitchell et al., 2015) and the BLASTP v2.10.1+ package (Ye et al., 2006) against Swiss-Prot (Boeckmann et al., 2003), NCBI non-redundant (nr), COG (Tatusov et al., 2003), and Kyoto Encyclopedia of Genes and Genomes (KEGG) (Kanehisa and Goto, 2000). The *E*-value cutoff was set to 1*e*-5 for BLAST searches.

### Comparative phylogenomic analysis

To identify homologous gene families in the yellowhorn genome, we performed a comparative genome analysis of yellowhorn and 16 other closely related angiosperm genomes (Supplementary Table 18) as previously described (Liang et al., 2019). After removing alternatively spliced transcripts and transposable elements, the most extended protein sequences (>50 amino acids) were collected. An all-vs-all comparison was performed using BLASTP with an *E*-value cutoff of 1*e*-5. The alignments were clustered into homologous groups using OrthoMCL v2.0.9 (Li et al., 2003). A maximum likelihood phylogenomic tree of the 17 species based on shared single-copy genes was constructed using RAxML v8.1.24 (Guindon et al., 2010), with parameters “-# 1000-m PROTGAMMALGX.” *O. satvia* was used as the outgroup. Species divergence times were estimated using the MCMCtree module in PAML v4.9 (Yang, 2007) based on three secondary calibration points (Supplementary Table 19) obtained from the TimeTree database^5^ (Hedges et al., 2006). The phylogenomic tree was visualized using FigTree v1.4.3 (Drummond and Rambaut, 2007).

Based on the inferred phylogenomic history, gene family expansion and contraction were detected using CAFÉ v4.2.1 (De Bie et al., 2006). The expanded and contracted gene families were annotated using HMMER v3.2.1 (Eddy, 2011) against the databases Pfam v32.0 (El-Gebali et al., 2019) and Swiss-Prot, respectively. The functional enrichment of each gene family was determined using a hypergeometric test (Cao and Zhang, 2014) under a *P*-value cutoff of 0.05.

### Analysis of ankyrin genes in yellowhorn

The HMMs of ankyrin (ANK) repeat protein clan PF00023 (Ank), PF12796 (Ank_2), PF00023 (Ank), PF13637 (Ank_4), and PF13857 (Ank_5) were used as queries to search against protein sequences of yellowhorn using HMMER v3.2.1 with *E*-value cut-off of 0.01. Multiple sequence alignments of the ANK protein sequences were performed using Clustal W (Larkin et al., 2007). The phylogenetic tree was established using RAxML software with 1000 bootstraps and exhibited in the EvolView online tool^6^ (He et al., 2016). In addition, 27 previous published transcriptomic datasets (Wang et al., 2020a,b) were obtained from the Sequence Read Archive (SRA) database in NCBI under accession number PRJNA608707 and used to investigate expression patterns of the ANK genes.

### Identification of lipid-associated genes in yellowhorn

A total of 684 lipid-associated genes of *A*. *thaliana* were obtained from the database of Arabidopsis Acyl-Lipid Metabolism (Li-Beisson et al., 2013), and used as query sequences to search against protein sequences of yellowhorn, *A. truncatum*, *M. oleifera*, *O. sativa*, *S. lycopersicum*, and *V. vinifera* using BLASTP v2.10.1 with parameters “*E*-value ≤ 1*e*-5, alignment identity ≥ 30%, and alignment coverage ≥ 50%.” For *long-chain acyl-CoA synthetase* (*LACS*) and *KCS* genes, the genes must have the same Pfam domains (PF13193 and PF13193 for *LACS*; PF08392 and PF08392 for *KCS*) as the query sequence.

### Kernel oil and fatty acid composition analysis

For oil extraction, kernels at 50, 56, 62, 68, 74, 80, and 86 DAF were collected and dried at 65°C until the samples attained a uniform weight. The oil was extracted and methylated as previously described (Fu et al., 2008; Yu et al., 2017). The fatty acid methyl esters were analyzed as previously described (Liang et al., 2021) using an Agilent 7890B gas chromatograph (Agilent Technologies, Little Falls, MN, United States), connected to a flame ionization detector with a DikmaCap DM-2560 Capillary Column (inner diameter 100 m × 0.25 mm; film thickness 0.2 μm). Supelco 37 Component FAME Mix (Supelco, Bellefonte, PA, United States) was used to identify the fatty acid methyl esters. Fatty acid peaks were identified by comparison to the retention times of the standards. Each peak was quantified based on peak area using HP3398A GC Chemstation software (Hewlett Packard, Santa Clara, CA, United States) compared with the standard. The determination was run in triplicate.

### Transcriptomic analysis for the biosynthesis of very long-chain unsaturated fatty acids

We analyzed the transcriptomes of kernels of the ‘Shanyou 1’ cultivar collected at 50, 56, 62, 68, and 74 DAF. Take three independent samples as biological replicates at each time point. Total RNA was isolated using TRIzol reagent (Thermo Fisher Scientific, Waltham, MA, United States) following the manufacturer’s instructions. RNA purity and integrity were respectively assessed using a Qubit 3.0 Fluorometer (Life Technologies, Carlsbad, CA, United States) and an Agilent Bioanalyzer 2100 with an RNA Nano 6000 Assay Kit (Agilent Technologies, Santa Clara, CA, United States). The RNA sequencing libraries were constructed using the TruSeq RNA Library Prep Kit v2 (Illumina, San Diego, CA, United States) following the manufacturer’s instructions and sequenced on an Illumina HiSeq 4000 platform (KeGene, Taian, China) with paired-end reads of 150 bp. Raw reads were first quality trimmed using Trimmomatic v0.39 (Bolger et al., 2014). The resulting high-quality clean reads were aligned to the yellowhorn genome assembly using HISTA2 v2.1.0 (Kim et al., 2015) and then assembled using StringTie v1.3.5 (Pertea et al., 2015). The gene expression was quantified with fragments per kilobase of exon model per million mapped fragments (FPKM). The genes that met FPKM > 1 in at least one sequencing library were retained for DEGs identification. The DEGs were analyzed using DESeq2 v1.30.0.^7^ Genes with *P* ≤ 0.05 and fold change ≥ 2.0 were considered significantly differentially expressed.

### Statistical analyses

We used R v4.0^8^ for statistical analysis. One-way analysis of variance with Tukey’s *post-hoc* test was used to identify statistically significant differences among groups. Data are expressed as the mean ± standard deviation (SD), and *P* ≤ 0.05 was considered a significant difference.

## Results

### Genome assembly and annotation

**FIGURE 1:**
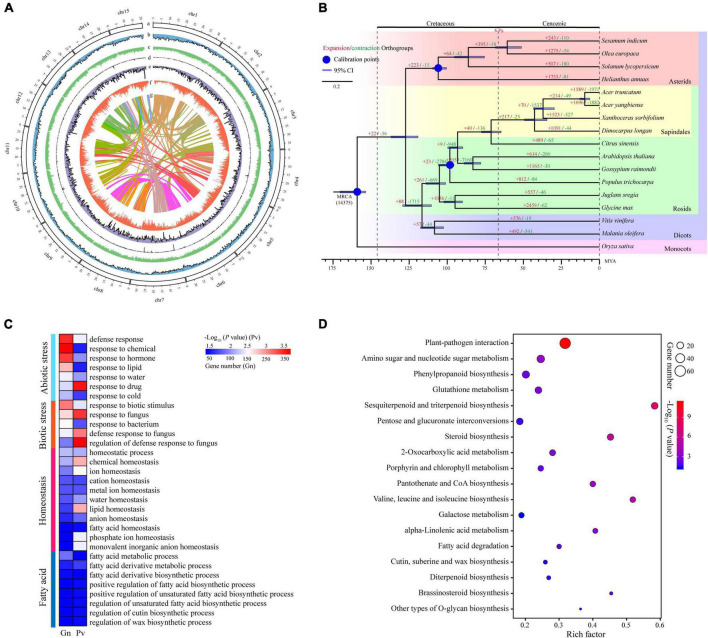
The characteristics of yellowhorn ‘Shanyou 1’ (WF18) genome assembly. **(A)** The distribution of genomic elements of yellowhorn. The items from outermost to innermost are: (a) pseudochromosomes, (b) LTR-Gypsy density, (c) LTR-Copia density, (d) transposon element density, (e) tandem repeat density, (f) gene density, and (g) intra-genome collinear blocks. Chromosome units correspond to Mbp. **(B)** Maximum-likelihood phylogeny of yellowhorn and 16 representative angiosperms, based on 223 shared single-copy genes. *Oryza sativa* was used as the outgroup. All nodes have 100% bootstrap support. Species divergence times were estimated using three secondary calibration points (blue circles) from the TimeTree database. Horizontal blue bars denote confidence intervals (CI). Values above the branches indicate the numbers of orthologous gene families that expanded (red) or contracted (green) after divergence from the most recent common ancestor (MRCA). K-Pg denotes the Cretaceous-Paleogene boundary. Abbreviation: MYA, millions of years ago. **(C)** The gene ontology (GO) categories are significantly enriched in the significantly expanded gene families. **(D)** The Kyoto Encyclopedia of Genes and Genomes (KEGG) pathways enriched the significantly expanded gene families.

We assembled the whole genome sequence of the yellowhorn cultivar ‘Shanyou 1’ (WF18). However, the genome assembly of this version still contains many gaps (1,000 gaps covering 29.06 Mb) (Liang et al., 2019). To enhance the accuracy and integrity, based on a combination of PacBio, Hi-C, and Illumina reads, we reassembled the whole genome sequence using a haplotype-resolved approach (Supplementary Tables 1, 2). By executing the FALCON-Unzip assembler with PacBio long reads, the initial assembly generated 2,428 primary contigs (N50 of 408.56 kb) with a total length of 482.89 Mb, as well as 5,310 haplotigs covering 264.58 Mb, accounted for 54.79% of the genome assembly (Supplementary Table 3). The primary contigs were further scaffolded using Hi-C reads to enhance the sequence continuity. As a result, a total length of 490.44 Mb genome sequence containing 22 super-scaffolds (N50 of 34.27 Mb) was generated, of which 490.24 Mb sequences accounting for 99.96% of the whole assembly were anchored onto 15 pseudochromosomes (Figure 1A and Supplementary Table 4). The size of the reassembled genome was more extensive than that of cultivar ‘JGXP’ (470 Mb) and our last assembly (440 Mb) but smaller than that of the cultivar ‘ZS4’ (504 Mb) (Table 1). Compared with our previous version, gap filling significantly improved the integrity of the assembly and left 2,295 gaps covering only 989.98 kb. Moreover, the mapping rates of Illumina and 98.81% PacBio reads were 98.62 and 98.81%, respectively; the single-base error rates of single nucleotide polymorphisms as well as insertion and deletions were, respectively, 0.000216 and 0.000421% (Supplementary Table 5). The complete BUSCOs recovery score of the assembly was 98.7% (1,594 of 1,614 genes) (Supplementary Table 6), which was much higher than our previous version (87.4%) (Table 1).

**TABLE 1:** Yellowhorn (*Xanthoceras sorbifolium*) genome assembly and annotation.

Type	Parameter	WF18 v2	WF18 v1	ZS4	JGXP
Assembly	Genome size (Mb)	490.44	439.97	504.2	470
	Chromosome-scale scaffolds (Mp)	490.24 (99.96%)	419.84 (95.42%)	489.29 (97.04%)	446.2 (94.9%)
	Total num. of scaffolds	22	267	2,297	988
	Total num. of chromosomes	15	15	15	15
	Scaffold N50 (Mb)	34.27	29.43	32.17	30.8
	Scaffold L50	7	7	8	–
	Total num. of contigs	2,428	2,002	3,035	3,302
	Contig N50 (Mb)	0.42	0.64	1.04	0.42
	Contig L50	237	291	131	–
	Complete BUSCOs	98.7%	87.4%	98.7%	97.5%
	GC content of the genome (%)	34.71	34.18	36.95	34.94
Annotation	Protein-coding genes	29,888	21,059	24,672	22,049
	Average gene length (bp)	4,600	7,040	4,199	4,277
	Average CDS length (bp)	1,335	1,337	1,580	1,326
	Average exon per transcript	7.39	15.61	–	6.06
	Total num. of tRNAs	776	–	642	708
	Total num. of rRNAs	64	–	316	65
	Total num. of miRNAs	41	–	–	–
	Total num. of snoRNAs	80	–	–	–
	Repeat sequences (bp)	290.68 (59.27%)	248.72 (56.39%)	346.36 (68.67%)	308.79 (65.7%)
	Annotated in Swiss-Prot	21,577	10,316	18,063	13,865
	Annotated in NCBI NR	26,263	13,315	24,316	20,944
	Annotated in COG	20,632	–	13,836	20,147
	Annotated in PFAM	20,895	16,838	20,795	18,357
	Annotated in GO	15,643	13,158	15,013	18,357
	Annotated in KEGG	8,917	3,423	8,599	9,002

Genome annotation identified 290.68 Mb repetitive sequences, occupying 59.27% of the genome assembly, of which 27.79% were long terminal repeat retrotransposon elements (Figure 1A and Supplementary Table 7). A total of 29,888 protein-coding genes were predicted (Table 1); 26,331 (88.10%) of them were annotated into at least one of the databases including NCBI NR (40.03%), Swiss-Prot (42.18%), protein families (Pfam) (69.91%), InterPro (72.19%), COG (42.27%), Gene Ontology (GO) (52.34%), and KEGG (28.18%).

### Gene family expansion analysis

Gene family expansion has been shown to facilitate adaptation and trait innovation in the evolution of angiosperms (Van de Peer et al., 2009; Wang et al., 2019; Ehrenreich, 2020). Therefore, to understand the genetic basis underpinning valuable fatty acids biosynthesis and environmental adaptability of yellowhorn, we analyzed gene family size using the phylogenomic tree. To this end, we first identified 223 single-copy nuclear genes conserved in 17 representative angiosperm genomes through comparative genomic analysis (Supplementary Table 8). The phylogenomic tree was then constructed using the conserved single-copy genes. The results suggested that the yellowhorn has diverged from a most recent common ancestor with *Dimocarpus longan* approximately 42.6 million years ago (Figure 1B and Supplementary Figure 1).

We next investigated the ancestral gene family size differences among species along the phylogenomic tree. The results showed that 1,523 gene families (e.g., *LACS* and *ANK* gene families) were significantly expanded (*P* < 0.01) while 327 ones were significantly contracted (Supplementary Table 9). GO functional enrichment analysis demonstrated that ten GO terms were related to fatty acid metabolic and biosynthetic processes, such as “fatty acid metabolic process,” “regulation of unsaturated fatty acid biosynthetic process,” and “regulation of cutin biosynthetic process.” Moreover, GO terms related to biotic or abiotic stress responses like “defense response,” “response to water,” “response to cold,” and “homeostatic process” were significantly enriched (*P* < 0.01) in the expanded gene families (Figure 1C and Supplementary Table 10). KEGG enrichment analysis also revealed that many expanded gene families were involved in fatty acid biosynthesis and stress adaptation, such as CoA biosynthesis (ko00770), plant-pathogen interaction (ko04626), and pantothenate (Figure 1D and Supplementary Table 11). Thus, these results suggested that gene family expansion was associated with fatty acid biosynthesis and stress adaptation in yellowhorn.

### Analysis of lipid-associated genes

**FIGURE 2:**
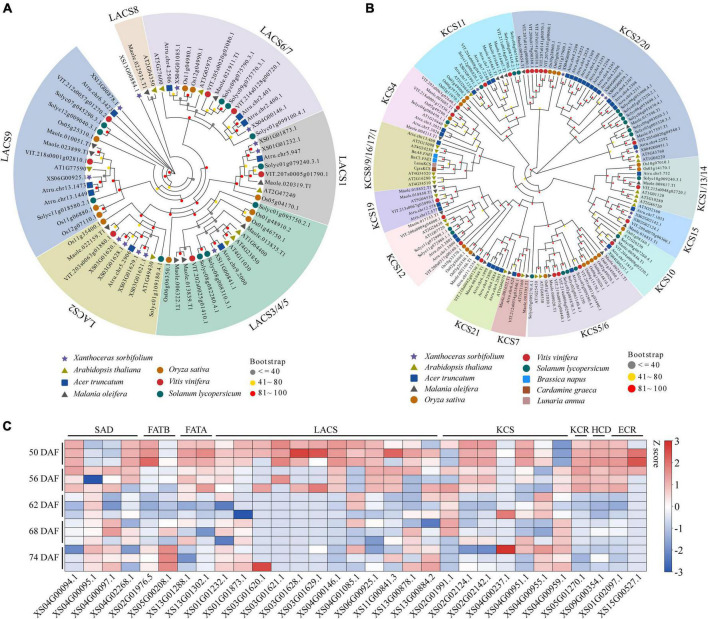
Gene expansion of LACS and KCS in yellowhorn. Maximum-likelihood phylogenetic tree showing expanded LACS **(A)** and KCS genes **(B)** in yellowhorn. The phylogenetic tree was constructed using RAxML software with 1000 bootstraps and exhibited in the EvolView online tool (https://www.evolgenius.info/evolview/). **(C)** Gene expression patterns of LACS, KCS, and other lipid-related genes among 15 kernel transcriptomes of 50, 56, 62, 68, and 74 DAF of yellowhorn.

To explore genes involved in VLCFAs biosynthesis in yellowhorn, especially nervonic acid and erucic acids, we blasted against the genome assembly using genes involved in lipid synthesis and metabolism from *A. thaliana* as the query sequences (196 functional families containing 684 genes). As a result, the 788 homologs were identified and designated as lipid-associated genes in yellowhorn. The number was much greater than that in *A. truncatum* (745) and *M. oleifera* (566), the other two VLCFAs-producing species, suggesting an active lipid biosynthesis and metabolism in yellowhorn (Supplementary Table 12). In the yellowhorn, lipid-associated gene families encoding LACS, ATP citrate lyase A subunit (ATP-CL-alpha), and alcohol-forming fatty acyl-CoA reductase (AlcFAR), which are essential for fatty acid synthesis, contained more members than those in *A. truncatum* and *M. oleifera*. The *LACS* family expanded to 12 members, and the four members were organized in a tandem array located in chromosome 3 (Supplementary Figure 2A). The phylogenetic tree showed that the *LACS* genes were clustered into six groups, while the LACS-2 group contained four tandem genes (Figure 2A). In addition, we found that the KCS family expanded to 8 members in the yellowhorn genome. Phylogenetic analysis showed that the *KCS* genes were clustered into four groups (Figure 2B). Four clustered *KCSs*, closely related to *KCS2* and *KCS20* in *A. thaliana*, were organized in a tandem array and located in an 86 kb region of chromosome 4 (Supplementary Figure 2B). Almost all the *LACS* and *KCS* genes expressed in a set of 15 yellowhorn kernel transcriptomes with FPKM ≥ 1 (Figure 2C), demonstrating their functional activities in erucic and nervonic acids biosynthesis. Therefore, we speculate that the expanded lipid-associated gene families, especially *LACS* and *KCS*, may play crucial roles in the yellowhorn biosynthesis of erucic and nervonic acids.

### Molecular basis of very long-chain unsaturated fatty acids biosynthesis

**FIGURE 3:**
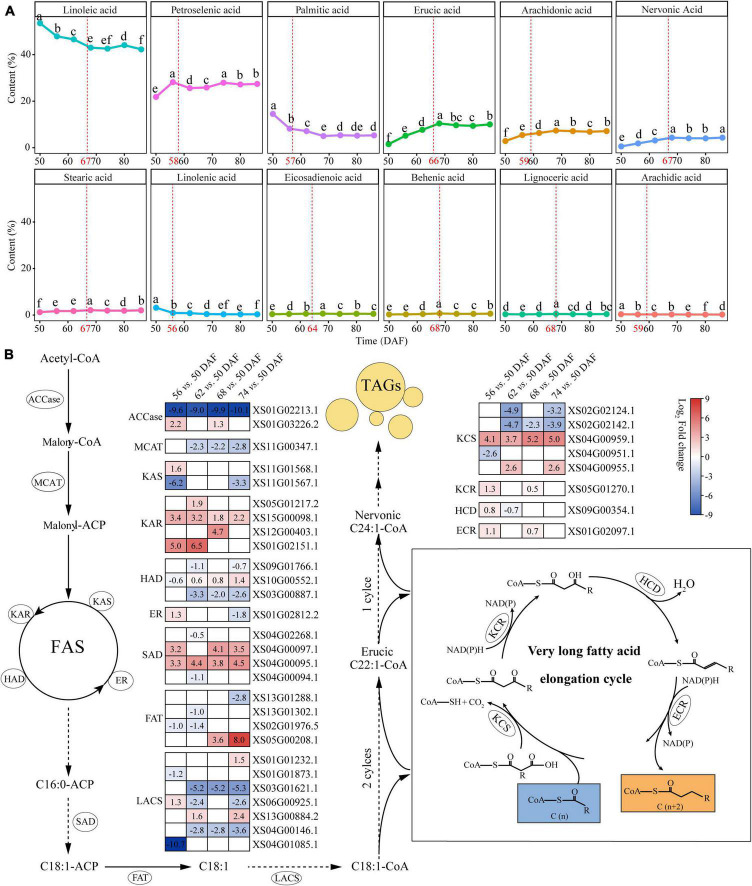
The biosynthetic pathways of nervonic and erucic acid in yellowhorn. **(A)** Fatty acid composition in yellowhorn seed oil at 50–86 DAF. Values shown are means ± SD of three biological replicates. The red dot plot represents the breakpoint analyzed using the breakpoint function of RStudio v4.0. Different letters on the dots indicate statistical significance (*P* ≤ 0.05). **(B)** The nervonic and erucic acid biosynthesis pathway, highlighting the differentially expressed with fold change ≥ 2 at 56, 62, 68, and 74 DAF compared with 50 DAF. Abbreviations: ACCase, acetyl-CoA carboxylase; MCMT, malonyl-CoA:acyl carrier protein (ACP) malonyltransferase; KAR, ketoacyl-ACP reductase; KAS, ketoacyl-ACP synthase; HAD, hydroxyacyl-ACP dehydrase; ER, enoyl-ACP reductase; SAD, stearoyl-ACP desaturase; FAT, fatty acyl thioesterase; LACS, long-chain acyl-CoA synthetase; KCS, ketoacyl-CoA synthase; KCR, ketoacyl-CoA reductase; HCD, hydroxyacyl-CoA dehydrase; ECR, enoyl-CoA reductase.

We performed profiles during kernel development from 50 to 86 DAF to investigate the characteristics of fatty acid composition in yellowhorn kernels. The seed oil accumulation increased rapidly from 50 to 68 DAF and reached content of 54.43% at 68 DAF (Supplementary Figure 3). Fatty acid profiling identified 12 components in kernels (Figure 3A and Supplementary Table 13). The results showed a difference in the content of different fatty acids at different time points (Figure 3A). Among these fatty acids, linoleic acid (42.24–53.48%) and oleic acid (21.73–28.08%) were the major components, whereas arachidic acid took the lowest proportion (0.20–0.34%). Nervonic and erucic acid accumulated significantly from 50 to 68 DAF and reached 4.07 and 10.41%, respectively.

Based on the nervonic and erucic acid accumulation during the yellowhorn kernel development period, the kernel at 50, 56, 62, 68, and 74 DAF were selected for transcriptomic analysis to elucidate the genetic mechanism underlying the biosynthesis of nervonic and erucic acid. Compared with 50 DAF, 165, 229, 212, and 247 differentially expressed lipid genes (DELGs) were identified at 56, 62, 68, and 74 DAF, respectively (Supplementary Figure 4 and Supplementary Table 14). Gene functional enrichment analysis showed that the DELGs were enriched in organic acid biosynthetic and metabolic processes, fatty acid biosynthesis and metabolism, biosynthesis of unsaturated fatty acids, and fatty acid elongation (Supplementary Figure 5 and Supplementary Table 15). Fifty-three members of the DELGs were involved in VLCFAs biosynthesis (Supplementary Table 16). Nervonic acid is biosynthesized from oleyl-CoA (C18:1-CoA) by four successive enzymatic reactions at the ER catalyzed by KCS, KCR, HCD, and ECR, respectively. The DELGs contained 5 *KCSs*, one *KCR*, one *HCD*, and one *ECR* (Figure 3B and Supplementary Table 16). Four genes were significantly upregulated (*P*-value < 0.05) at 56, 62, 68, and 74 DAF compared with 50 DAF. Notably, the transcription level of *XS04G00959*, which encodes one KCS, was highly expressed at 56 (17.15-fold), 62 (13.00-fold), 68 (36.76-fold), and 74 (32.00-fold) DAF compared with 50 DAF, which was consistent with the accumulation of nervonic and erucic acid (Figure 3B and Supplementary Table 16), suggesting that *XS04G00959* played essential roles in the biosynthesis of nervonic and erucic acid.

### Analysis of ankyrin repeat gene family

**FIGURE 4:**
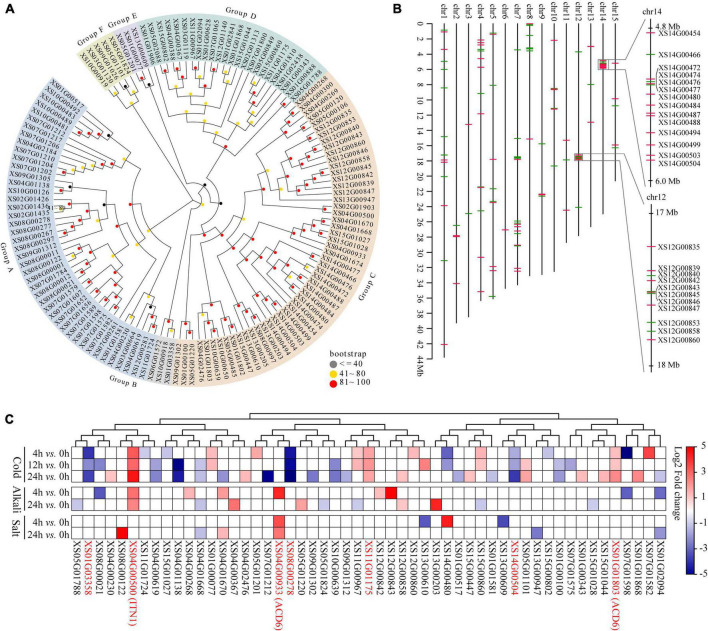
Gene expansion of ANK in yellowhorn. **(A)** Maximum-likelihood phylogenetic tree showing expanded ANK in yellowhorn. The phylogenetic tree was established using RAxML software with 1000 bootstraps and exhibited in the EvolView online tool (https://www.evolgenius.info/evolview/). **(B)** Distribution of the 132 ANK genes along 15 yellowhorn pseudochromosomes. The right side shows the detailed overview of ANK gene clusters on pseudochromosomes 12 (from 17 to 18 Mb) and 14 (from 4.8 to 6.0 Mb), respectively. The red and green lines represent the genes located on the senesce or antisense strand of the pseudochromosome. **(C)** The 53 significantly differentially expressed ANK genes were identified after cold, salt, and saline-alkali stress. The 27 transcriptomic datasets were downloaded from the NCBI Sequence Read Archive (SRA) database under accession PRJNA608707.

*ANK* genes play crucial roles in biotic or abiotic stress responses (Becerra et al., 2004; Huang et al., 2009), the most prominent expanded gene families in yellowhorn genome assembly. To explore the potential roles of the *ANK* family in environmental adaptation in yellowhorn, the characteristics of *ANK* genes were analyzed. The results showed that the *ANKs* family in yellowhorn expanded to 132 members, which were further clustered into six distinct groups based on phylogenetic analysis (Figure 4A). The *ANK* genes were distributed across all the 15 pseudochromosomes, with 14 and 11 genes organized in tandem arrays on pseudochromosome 14 and 12 (Figure 4B). The previous published transcriptomic datasets were re-analyzed to determine the expression patterns of *ANKs* responding to abiotic stress. The results indicated that 53 *ANK* genes were significantly differentially expressed under cold, salt, or saline-alkali stresses (Figure 4C and Supplementary Table 17). Of them, six members, including *XS01G01803* [*accelerated cell death 6* (*ACD6*)], *XS04G00500* [*ankyrin repeat-containing protein ITN1* (*ITN1*)], *XS14G00504*, *XS11G01175*, *XS08G00278*, and *XS01G03358* were significantly differentially expressed under cold stress. *XS04G00933* (*ACD6*) was significantly upregulated after 4 and 24 h exposure to salt stress. Both cold and saline-alkali stresses significantly induced the expression of *XS04G00500* (*ITN1*). These results suggested that *ACD6* and *ITN1* were related to different abiotic stresses response in yellowhorn.

## Discussion

High-quality genome assembly is of vital importance for biological studies and breeding applications. The yellowhorn genome assemblies have been reported previously by other teams and us (Bi et al., 2019; Liang et al., 2019; Liu H. et al., 2021). This study reconstructed the yellowhorn cultivar ‘Shanyou 1’ genome at the haplotype-resolved level based on PacBio, Hi-C, and Illumina reads (Chin et al., 2016). Compared with the previous versions, the percentage of contig anchored to the pseudochromosomes, N50 length, and complete BUSCOs were significantly increased. More than 99% of the contigs can be anchored to the pseudochromosomes, 98.7% of BUSCO genes were complete, and more than 99% of the gaps were closed. Therefore, the reassembled genome sequence is of high completeness, accuracy, and continuity and can be used as a reference for studies on genome function and genetic variation.

Our results revealed that *LACS*, *ATP-CL-alpha*, and *AlcFAR* families, which are responsible for fatty acid synthesis, contained more members than those in other VLCFA-producing woody plants, such as *A. truncatum* and *M. oleifera*. The *LACS* family genes have been shown to participate in fatty acid and glycerolipid metabolism (Lü et al., 2009; Zhao et al., 2010, 2019; Jessen et al., 2015). In *Brassica napus*, *LACS2* plays a crucial role in seed oil production (Ding et al., 2020). *Arabidopsis LACS4* and *LACS9* are involved in the biosynthesis of glycolipids in plastids (Jessen et al., 2015). In the present study, we found that the *LACS* family expanded to 12 members in yellowhorn. Most of these genes were preferentially expressed at 56 and 62 DAF, suggesting their roles in producing fatty acyl supply flux for fatty acid synthesis (Lin et al., 2018). Moreover, the *KCS* family expanded to eight members, which fell into four major clades. It has been shown that ectopic expression of *Lunaria* and *Cardamine KCSs* in *Brassica* increased nervonic acid and reduced erucic acid accumulation in the seed oil (Guo et al., 2009; Taylor et al., 2009). Our results showed that two *KCSs* were significantly upregulated at 56 and 62 DAF, which is consistent with the accumulating stage of nervonic acids, suggesting their regulatory roles in this process.

Recent studies revealed that KCS also demonstrates strong substrate specificity, which defines the final chain length of the VLCFAs (Liu F. et al., 2021). Our analysis revealed that the accumulation of fatty acids and biosynthesis of both nervonic and erucic acid were mainly completed between 50 and 68 DAF. Transcriptomic analysis demonstrated that 36 genes regulating VLCFA elongation were differently expressed at 56, 62, 68, and 74 DAF compared with 50 DAF. *XS04G00959* encoding KCS was significantly upregulated and exhibited an expression trend consistent with the accumulation of nervonic and erucic acid, suggesting that this gene is essential for the biosynthesis of nervonic and erucic acid.

Substantial expansions were identified in gene families associated with stress responses. In the present study, we found that the *ANK* family expanded significantly and contained 132 members. Several *ANK* genes have been shown to resist biotic or abiotic stresses (Becerra et al., 2004; Huang et al., 2009). For instance, *ACD6* participates in salicylic acid signaling in *Arabidopsis*, and overexpressing this gene enhances pathogen resistance (Lu et al., 2003; Zhang et al., 2014). *ITN1* mediates responses to salt stress by promoting the production of reactive oxygen species *via* the abscisic acid signaling pathway (Sakamoto et al., 2008, 2012). Based on published transcriptomic data, we found that transcription of two *ACD6* copies (*XS04G00933* and *XS01G01803*) was significantly induced by salt and cold stresses. Both cold and saline-alkali stresses significantly upregulated the *ITN1* homolog (*XS04G00500*). These results suggested that the expansion of *ANK* family may contribute to the resistance against abiotic stresses in yellowhorn.

## Conclusion

In summary, we reported a haplotype-resolved chromosome-scale genome assembly of yellowhorn. We identified one *KCS* gene, *XS04G00959*, which can be a vital contributor to the nervonic and erucic acid biosynthesis in yellowhorn. We revealed that *ACD6* and *ITN1* played crucial roles in yellowhorn against multiple abiotic stresses. Altogether, the present study provides new insights into the understanding of the economic traits such as valuable fatty acid production and environmental adaptation, thus greatly benefiting the genetic improvement of this species.

## Data availability statement

The original contributions presented in this study are publicly available. This data can be found here: NCBI, PRJNA723435.

## Author contributions

KY and YS designed and supervised the project and reviewed and revised the manuscript. QL and JL collected and generated the data, performed analysis, and wrote the draft manuscript. YD, RZ, and XM performed transcriptome analysis. HF, RZ, XM, and CW contributed to data analysis. CW, YB, WH, SG, LW, and SL contributed to plant material collection. All authors contributed to the article and approved the submitted version.

## Footnotes

1
https://slps.jdzx.net.cn/xwfb/gzcx/PassFileQuery.jsp

2
https://github.com/PacificBiosciences/FALCON_unzip/

3
https://github.com/PacificBiosciences/pbbioconda/

4
http://www.repeatmasker.org/RepeatModeler/

5
http://www.timetree.org/

6
https://www.evolgenius.info/evolview/

7
https://bioconductor.org/packages/DESeq2/

8
https://www.r-project.org/
