# Supplementary material for: Genomic and transcriptomic analyses provide insights into valuable fatty acid biosynthesis and environmental adaptation of yellowhorn
Source: Front Plant Sci. 2022 Sep 6;13:991197. doi: 10.3389/fpls.2022.991197 (PMC9486082; doi:10.3389/fpls.2022.991197)
Supplement: Supplementary file 1 [file Data_Sheet_1.docx]

Supplementary Material

1. **Supplementary Figures and Tables**
   1. **Supplementary Figures**


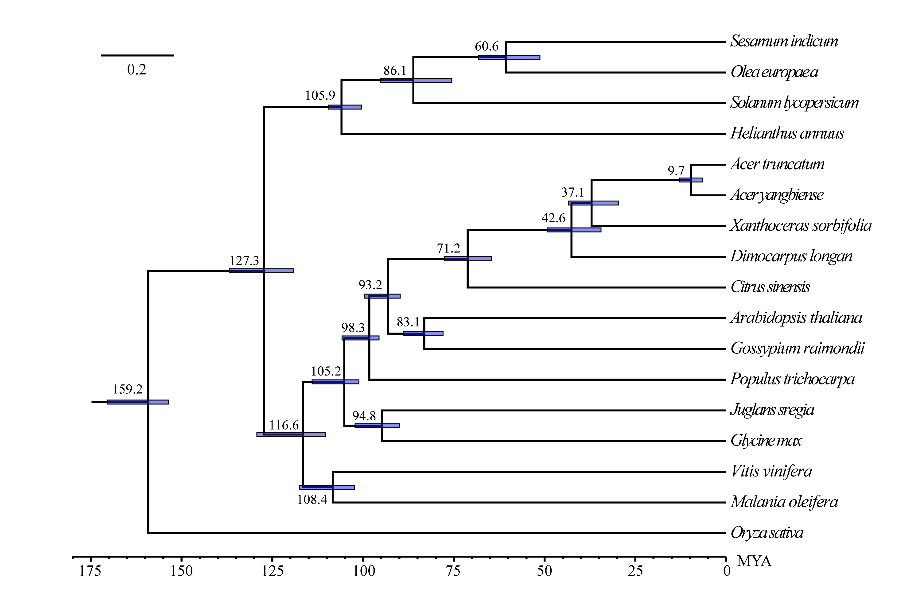


**Supplementary Figure 1.** Phylogenetic tree of yellowhorn and 16 representative angiosperms. The tree was constructed using RAxML software based on 223 shared single-copy genes. The bootstrap support value of each node is 100. The values above the branches indicate the predicted divergence time. The light blue bars indicate 95% of confidence interval score. MYA, million years ago.


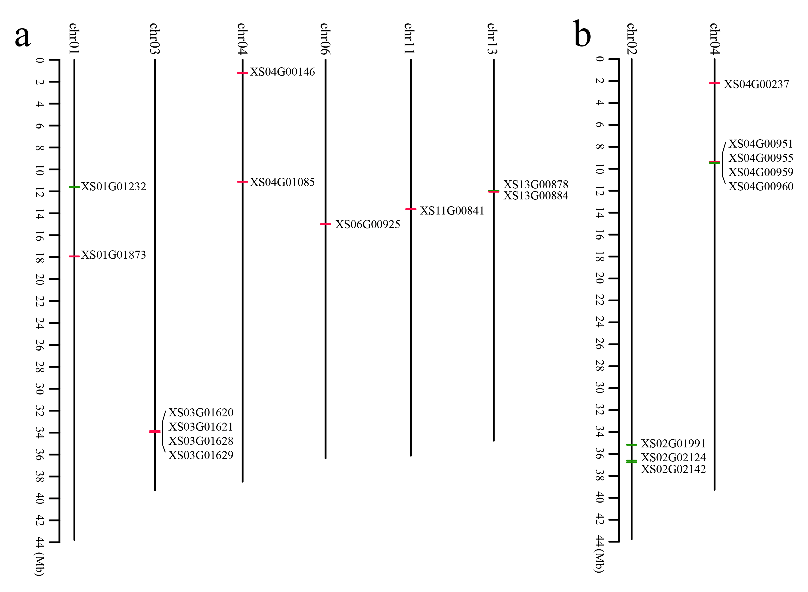


**Supplementary Figure 2.** Distribution of the 12 LACS genes (a) and 8 KCS genes (b) along 15 yellowhorn pseudochromosomes.


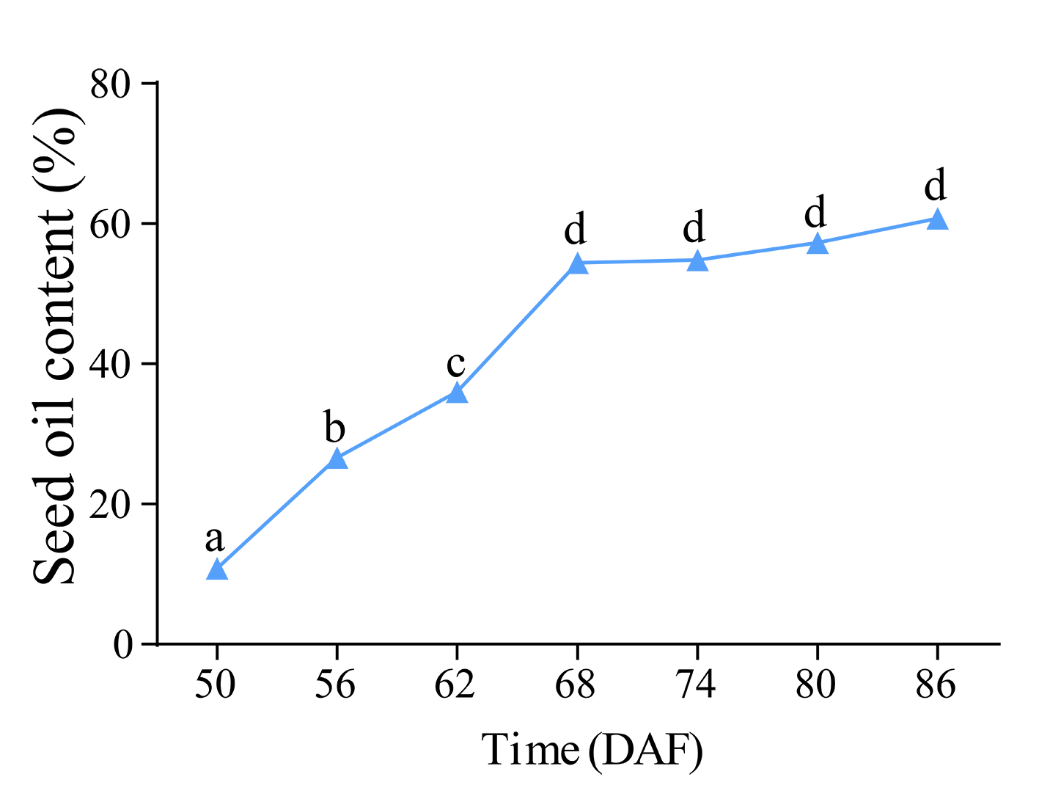


**Supplementary Figure 3.** Seed oil content in yellowhorn seeds after flowering. Data of seed oil content are the means ± standard deviations (SD) of 5 individual fruits of yellowhorn. Different letters indicate significant differences at P ≤ 0.05 by one-way ANOVA analysis with post-hoc test. DAF, days after flowering.


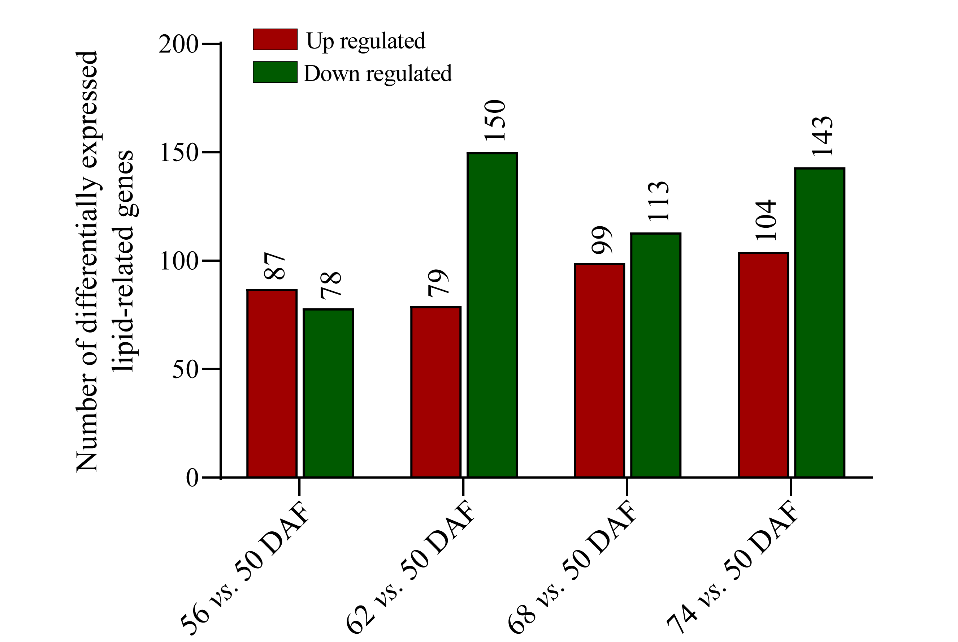


**Supplementary Figure 4.** Numbers of differentially expressed lipid-associated genes at 56, 62, 68, and 74 DAF compared with 50 DAF.


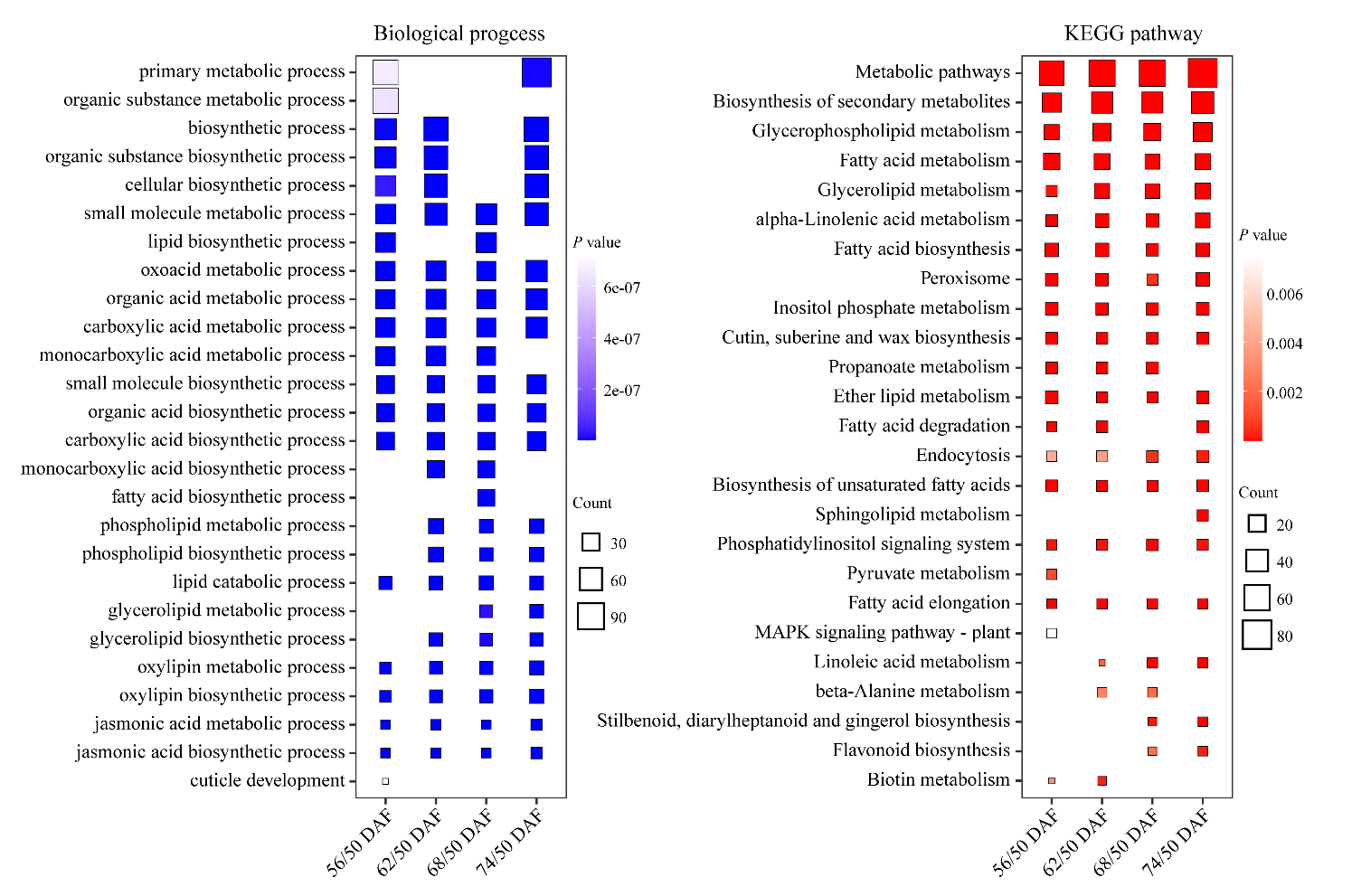


**Supplementary Figure 5.** Gene functional enrichment analysis of differentially expressed lipid-associated genes at 56, 62, 68, and 74 DAF compared with 50 DAF.

## Supplementary Tables

**Supplementary Table 1.** Statistics of Illumina short read sequencing data

**Supplementary Table 2.** Statistics of PacBio long read sequencing data

**Supplementary Table 3.** Summary of initial yellowhorn genome assembly by FALCON-Unzip assembler

**Supplementary Table 4.** Detailed information of the pseudochromosomes anchored with Hi-C

**Supplementary Table 5.** Statistic of variants in yellowhorn genome assembly

**Supplementary Table 6.** Statistic of the completeness of yellowhorn genome assembly

**Supplementary Table 7.** Repeat elements of yellowhorn genome assembly

**Supplementary Table 8.** Shared single-copy nuclear genes between yellowhorn and other 16 representative angiosperm genomes

**Supplementary Table 9.** The significantly expanded orthologous gene families among 17 angiosperms

**Supplementary Table 10.** Gene ontology (GO) enrichment analysis of the significantly expanded orthologous gene families of yellowhorn

**Supplementary Table 11.** KEGG pathway enrichment analysis of the significantly expanded orthologous gene families of yellowhorn

**Supplementary Table 12.** The lipid-associated genes in yellowhorn genome assembly

**Supplementary Table 13.** Fatty acid profiles of yellowhorn seeds after flowering

**Supplementary Table 14.** Differentially expressed lipid-related genes at 56, 62, 68, and 74 DAF compared with 50 DAF

**Supplementary Table 15.** Gene ontology (GO) enrichment analysis of the differentially expressed lipid genes

**Supplementary Table 16.** The putative genes involved in very long-chain monounsaturated fatty acids biosynthesis pathway

**Supplementary Table 17.** The significantly differentially expressed ANK genes under cold, salt, and saline-alkali stress

**Supplementary Table 18.** The genome information of 16 plant species

**Supplementary Table 19.** The secondary calibration points from TimeTree website (http://www.timetree.org/)
